# Extended home visits from child healthcare nurses and dental nurses: an interview study with first-time parents participating in the home visit program in southern Sweden

**DOI:** 10.3389/fpubh.2025.1609363

**Published:** 2026-01-14

**Authors:** Karin Persson, Susanne Brogårdh-Roth, Elisabeth Mangrio

**Affiliations:** 1 Department of Care Science, Malmö University, Malmö, Sweden; 2 Department of Pediatric Dentistry, Faculty of Odontology, Malmö University, Malmö, Sweden

**Keywords:** child health care, infants, dental care, home visit program, qualitative

## Abstract

**Introduction:**

This study is a part of a series of studies on a health and social care home-visit program in Sweden called Grow Safely. The program aimed to reduce health disparities in underprivileged areas. The current study evaluated home visits from Child Health Care and dental services that took place when the children were 8 months old and illuminated their parents’ experiences of the home visits from both pediatric nurses and dental nurses.

**Methods:**

Interviews were conducted with 18 first-time families after they received the home visits. The interviews were analyzed using Burnard’s approach to content analysis. Ethical approval from the Regional Ethical Committee in Lund, Sweden, was obtained before the study was conducted.

**Results:**

The results included three categories. The parents appreciated the home visits since both they and their baby felt comfortable and secure during the visits. They found the information about their child’s development and the advice on good oral health valuable. They also valued the tailored information on how to ensure that their home was safe and secure for the child.

**Conclusion:**

The first-time parents appreciated the extended home visits when the baby was 8 months old. Further studies are needed in order to evaluate the effect of these visits on oral health.

## Introduction

The Swedish child health care (CHC) is a program funded by the county councils and regions that conducts regular check-ups of children aged 0–6 years to follow closely the child’s physical, mental, and social development and support parents in their parenting (1). In addition, the CHC offers parents two home visits by a CHC nurse, one directly after birth and one when the child is 8 months old (2). Almost all children in Sweden (99%) receive this government funded child health care (1). Home visits offered to infants and their parents have shown good outcomes in earlier studies that considered both mental and physical health (3–6). Moreover, a previous Swedish study on experiences among foreign-born parents and their encounters with the CHC in Sweden showed that several of the interviewed parents emphasized the importance of the home visits but also how they appreciated the program (7). In general, families with a migration background tend to have high levels of trust in Swedish child healthcare (8), suggesting that the system is effective in reaching and serving all families, including those from diverse backgrounds.

One extended CHC home-visit program launched in 2013 in a disadvantaged area in Stockholm offered all first-time parents six home visits instead of two, and a parent advisor joined the CHC nurse for all the visits (9). Studies on the program found that vaccination rates increased and hospital visits decreased (10) and that trust was built between the healthcare professionals and the parents (11). Inspired by this home-visit program, several other programs started throughout Sweden, including Grow Safely, which is the focus of the current study (12). Grow Safely took place between 2019 and 2023 and was funded by Region Scania and the Swedish Association of Local Authorities and Regions with the aim of contributing to equal health among all children in Sweden. The whole county of Scania was included in the intervention, and all CHC centers in the county could volunteer for participation and through this universal intervention compared to the more targeted intervention in Stockholm, all families including diverse ones were reached. Grow Safely included six home visits for first-time parents that were conducted when the child was 0–15 months old, and in addition to the CHC nurses, the home visits were carried out by midwives, parent advisors, and dental nurses (12). The aspect of Grow Safely that differed from the program in Stockholm was the involvement of both midwives and dental nurses. The dental nurse training program is a three-semester course offered at a vocational college that prepares students to assist dentists during treatments, deliver preventive dental information, and support patients during their visits.

In addition to mental and physical health, the early intervention in Grow Safely could also benefit oral health. Oral health is fundamental for an overall good health and quality of life. Although the Swedish dental care system has a strategy to identify children at risk for caries at an early age and to improve dietary habits, oral hygiene, and the use of fluoride toothpaste, children get caries anyway. Early childhood caries (ECC) may influence children’s wellbeing. In particular, ECC may lead to pain, chronic dental infection, and inflammation; disrupt children’s sleep; and reduce their chewing ability, which could lead to malnutrition and growth problems (13). Importantly, ECC may develop rapidly because the enamel of primary teeth is thinner than that of permanent teeth (14, 15). This necessitates a preventive approach directed at improving not only the parents’ level of knowledge but also their attitude to health interventions, including oral health.

In addition, the collaboration within Grow Safely between child healthcare nurses, social services, maternity care, and dental care could contribute to reducing the differences in health between population groups in Sweden. Early interdisciplinary efforts in Grow Safely aimed to reduce the existing health disparities and to benefit health, including good oral health, in all children. Therefore, it is of importance to illuminate the experiences of parents who have participated in the project and received home visits from both pediatric nurses and dental nurses when their baby was 8 months old and how they perceived these visits.

## Method

The current study on the early intervention program Grow Safely is part of a research project along with several earlier published studies (12, 16, 17). It focused on the experiences of first-time parents who participated in the home-visit program when their child was at the age of 8 months and where the CHC nurses were joined by dental nurses. Since the goal was to illuminate the experience of undergoing these visits, a qualitative study was chosen. Qualitative research is ideal when a researcher aims to capture participants’ subjective perspectives on a phenomenon rather than produce generalizable insights about large groups. For example, qualitative interviews are particularly effective for exploring personal experiences (18). The data were collected through interviews, and all authors had prior experience in conducting interview studies. The current study employed an inductive content analysis approach to data analysis. This means that the data was examined with little or no reliance on a predetermined theory, structure, or framework, allowing the analytical structure to emerge directly from the data (19).

### Data collection

The recruitment of informants among parents was done by the CHC nurses working at the CHC centers that were involved in Grow Safely. The CHC nurses gave parents written information about the study, and this information was translated into 10 different languages to ensure families with foreign backgrounds could participate. Whenever the CHC nurses found a family that was willing to participate in the interviews, they emailed the research leader of Grow Safely; thereafter, researchers from the research group emailed or called the family to provide more information about the study. Thus, the sampling was considered a convenience sampling. The authors had no prior contact with the families. A total of 20 families accepted to take part in the interviews, but two of them were unreachable on the date of their interviews. Therefore, the study included 18 families. One interview was performed with both parents present, and the rest had only one parent present (19 parents in total). The parents were between 21 and 39 years of age. In 11 out of 18 families, both parents were born in Sweden, and in the rest, one or both parents were born abroad. Individuals born outside Sweden originated from Bosnia, Lebanon, Egypt, Iraq, Canada, and Croatia. Of the 19 parents, 12 had a university degree, 6 had a high school degree, and 1 had elementary school education. The parents lived in both rural and urban areas.

Due to the restrictions during the COVID-19 pandemic, all interviews were completed by telephone. Four families did not receive an 8-month home visit from the dental nurse; instead, they were visited only by the child healthcare nurse. Two interviews were held in English and the rest in Swedish. The interviews lasted between 10 and 26 min, and the total interview time was 259 min. All interviews were done by the first and second authors, and they were both present during all interviews. The interviews were carried out according to an interview guide (see Table 1), and they were recorded and transcribed shortly after each interview. Since the interview guide was quite broad, only a select subset of questions was chosen for the analysis in this study (marked in bold Table 1). After the 18 interviews were conducted, the data were saturated since no new information appeared and similar answers appeared during the last interviews and it was therefore considered saturated, and a decision was made to not collect more data after the 18th interview. No transcripts of the interviews were shown to the informants.

**Table 1:** Interview guide (the questions in bold were the focus for the analysis of the current study).

Interview questions
What expectations did you have of child healthcare before you entered the home-visit project? What made you agree to participate in the project? Can you talk about your experiences of participating in the project? **How did you experience getting a home visit by the child healthcare nurse and the dental nurse?** **How did you experience getting a home visit from two different healthcare professionals?** **Did you miss anything during the home visits?** What impact has the project had on your parental ability? How would you like to get support from the child healthcare center in the near future?

### Data analysis

After the interviews were transcribed, the written material was coded by the first author in relation to the aim of the study, and the coding was checked by the second and last authors. Thereafter, the codes that were similar were grouped together and formed three categories. Subsequently, the steps of the analysis were performed following Burnard’s method of content analysis (19). No software tool was used for the analysis, and none of the informants checked the coding. An example of the coding and analysis of the results is presented in Table 2.

**Table 2:** Coding schedule from meaning unit to code to sub-category.

Meaning unit	Code	Sub-category
And we talked about caries and how important it is to avoid them, and that they can… well, how should I put it, sort of “spread” to the permanent teeth, and that you should avoid sugar—even the kind you might not think about in products without added sugar.	It’s important to protect the teeth and that you should avoid products with sugar	The extended support increased the parents’ sense of confidence in dental care
The nurse talked about safety in the apartment and whether the child would fall and hit her teeth and whether it would bleed much and what we are supposed to do with our child.	Talked about safety and how to deal with a dental injury	Advantages of having safety information during the home-visits

## Results

The analysis of the data resulted in three categories: “*the extended support increased the parents’ sense of confidence in dental care*” *“Some parents raised concerns about the home-visit with the dental nurse*” and “*Advantages of having safety information during the home-visits*”

### The extended support increased the parents’ sense of confidence in dental care

The informants perceived the information they received on the home-visit occasions to be adapted to their current situation. The home visit at 8 months, coincided with the children having just had their first tooth, and information about oral health was seen as very timely. The informants appreciated the possibility to directly get answers to the questions that had arisen in connection with such circumstance from the dental nurses. They also received dietary advice and information about the importance of starting early with tooth brushing. During the eight-month visit, it was customary to inform parents that every child in Sweden is entitled to free dental care and that they can choose their own healthcare provider. Furthermore, one parent talked about dental information as follows:

It was quite nice. They came here and we began with all the tooth things, because H had just started to get his first tooth, so we talked a little about what good timing it was, and then we went over to the subject of tooth brushing, how it works, and things like that. (Informant 5, father)

Other parents appreciated receiving information that recommended starting to brush their child’s teeth at this age in order to establish good oral hygiene habits and prevent early childhood caries (ECC). They were also provided with guidance on avoiding sugar-containing products. In addition, there were discussions about how many times per day the teeth should be brushed; one parent commented:

"I was brushing once, and she told me that I need to brush twice" (Informant 15, mother).

Furthermore, parents were advised to brush their child’s teeth in the evening after the last meal rather than before eating. Overall, the information provided during the visits was perceived as building the parents’ confidence.

### Some parents raised concerns about the home-visit with the dental nurse

The interviews revealed that not all of the healthcare professionals were used to encountering questions that were directly related to the children’s age and development at the time of the visit, something at which the informants voiced their disappointment and the disappointment was related to the dental nurse.

One parent said like this:

“And regarding the dental part, I was thinking that maybe, if this is something that continues, it could be adapted a bit depending on when the child… children get their teeth at such different times” (Informant 16)

In addition, the informants expressed expecting the visits to be carried out by professionals with a connection to the geographical area that the informants came from. Consequently, they also criticized the fact that the dental nurses were employed by private actors without any connection to “their” CHC.

Moreover, the informants stated that they had expected all the different professionals participating in the project to visit them, and they expressed disappointment in cases where this did not happen. Nevertheless, the interviews revealed that it was mainly visits by professionals from dental care that had not taken place. This may have been related to rescheduling due to illness among both the families and the staff within the healthcare organizations engaged in the project, as the study was performed during the COVID-19 pandemic. One parent raised a concern when the dental nurse showed images of teeth with severe cavities. The parent remarked,

"Caries in the teeth —yes, she showed pictures and said that if you don’t brush your child’s teeth, they could look like this," which left her quite frightened. (Informant 17 mother)

Several parents felt that the dental nurse should have placed more emphasis on thoroughly examining their child’s mouth and teeth. Some even had higher expectations regarding the level of care provided, suggesting that the dental care information should be tailored to the specific needs and circumstances of the parents at the time of the visit.

### Advantages of having safety information during the home-visits

Participation in the project was mainly motivated by the parents’ need to ensure their children’s safety and to create the optimal possibility for their development. Several informants were keen to stress that they had joined the project because this would mean increased safety for the child.

The informants saw the home visits as valuable because they assumed that the different professionals were knowledgeable about and prioritized the child and the child’s safety. Several informants stated that they had been wondering about safety issues in their home, so they appreciated the opportunity to discuss these issues in their home environment. Namely, the setting of the visits made it possible for them to receive advice about how to avoid child accidents, advice that was adapted to the home environment in question; this concerned everything from how to store chemicals to what to do if the child injured their teeth. Reflections about the visits leading to thoughts about one’s parenting capacity being scrutinized were mentioned during the interviews. However, the informants dismissed such reflections during the visits since they were met by openness and kindness from the professionals, who had suggestions for adapting the home environment to make it a safe place for the child, both physically and mentally. That is, their advice concerned not only the physical environment but also the ways in which the parents could interact with their child in the home environment.

I mean, the nurse comes to my apartment and checks if it suits my child, if it is safe for him or not. […] Yes, so that I can feel at ease. She knows a lot about … she knows many things, more than I anyway. She has experience and such, so that’s what I like most. (Informant 14 mother)

Parents also appreciated the safety information provided for managing dental injuries. For example, if their child were to fall or had a dental injury, they were advised to contact emergency dental services immediately. Additionally, they received guidance on handling warm drinks and food safely around their young child to prevent burn injuries.

## Discussion

In sum, the results showed that the parents valued getting visits by two different healthcare professionals who could provide expert information and support based on their area of expertise. The parents also expressed being thankful for the information that they received around safety and security in the home environment. In particular, they were grateful for the practical instructions from both the child healthcare nurse and the dental nurse about how to secure the home as well as handle dental trauma and they were thankful that this visit took place in their own home. Some concerns were raised that some families did not receive the 8-month home visit from the dental nurse but only from the child healthcare nurse. Additionally, some parents expressed a desire for a more thorough examination of their child’s mouth and teeth.

The finding that the parents in the current study appreciated the visits taking place in their homes is confirmed by another study conducted a few years ago among immigrant parents in Sweden and their experience with the child health care: They highlighted their appreciation as first-time parents for receiving home visits by the CHC providers and felt such respect for them coming and visiting them in their homes (7). Another study within the same research project that illuminated the experience of getting home visits by both the CHC nurse and the midwife at the same time also confirmed this finding (16). But also another Swedish study confirmed that home-visits were appreciated within the homes since it felt like a safe place for them (20), and another Swedish study could see that it had advantages from both a logistical and practical point of view (21). The importance of home visits became especially evident during the pandemic, particularly in reaching vulnerable populations who needed support and access to health professionals without leaving home (22). Although families with diverse and migration backgrounds were included in this universal intervention, the aim of the current study was to explore the perspectives of all participating families, not to focus specifically on those groups. Nonetheless, it is important to examine how interventions like Grow Safely address the unique challenges faced by families with migration backgrounds or other forms of diversity. In this study, representation from families with different migration backgrounds was limited, and although some participants may have reflected diverse perspectives, no quotes or data clearly highlighted these experiences.

The parents also expressed appreciation for the two different professionals, the CHC nurse and the dental nurse, visiting them at the same time and providing different information and support based on their role and background. Collaboration between different professionals is essential when there is a need to create a holistic view for families in need of support (23). Interprofessional collaboration is also necessary to provide quality healthcare in today’s complex world and to manage the challenges that healthcare systems face (24). Moreover, such collaboration ensures that families receive a wide array of knowledge and skills that can only be acquired from different healthcare professions. When the baby is eight months old, the CHC nurse is supposed to give not only all the ordinary information about nutrition, sleep, safety, and development but also information about oral health. Therefore, it is important for the CHC nurse to get input from a professional who has expertise within oral health in order to provide better support for the families in need of establishing good oral health behavior as an early intervention to reduce the likelihood of ECC (25). Early intervention is important because ECC is a strong predictor of caries later in life (13–15, 25). However, some parents raised concern about the information and support from the dental nurse and that they wished for more adapted information related to the age of the child. This concern could be related to dental nurses coming from a private company mostly engaged with older people in society and it would be better with the public dental healthcare being involved, since they are more used to working with younger children and infants.

The results also showed that the parents liked the information they received about ensuring security in the home and preventing the child from getting injuries at home, such as burns and fall-related injuries. A study from Pakistan investigated an intervention through home visits for parents with children below three years of age and found a reduction of injuries because of the intervention (26). Therefore, the current intervention could be beneficial in preventing home injuries among small children and prevent dental injuries.

### Strengths and limitations

The study includes a relatively high number of interviews (N = 18), although they were relatively short. When it comes to ethnicity, 11 out of 18 families had both parents born in Sweden, but the rest had either one or both parents born abroad, which increased the diversity of the parents’ background in the study. Since the current study took place during the COVID-19 pandemic, the interviews had to be conducted through telephone, which could hold both advantages and disadvantages. Telephone interviews arguably have a lower risk for power imbalance compared to face-face interviews, which could make them an advantage (27). Another advantage to telephone interviews is that they allowed us to reach informants more conveniently wherever they are and that they allow the informants to be comfortable in their homes when doing the interviews. On the other hand, we were not able to see the informants in person, which could be a disadvantage, especially when sensitive issues are discussed (28). Another limitation of the present study was that, out of 18 families, four received a visit only from the child healthcare nurse rather than from the dental nurse. This discrepancy may have influenced the results as well as the parents’ perceptions of these visits. The study was analyzed using Burnard’s method of content analysis, and there was a continuous discussion among the authors during the analysis process, which was a strength of the study. In addition, we used a coding schedule, which strengthens the transparency and reliability of the analysis. When describing the procedure, we included quotations from the informants to enable the readers to determine the trustworthiness of the study (29). Even if the intervention was universal—spanning both urban and rural areas across various socio-economic contexts— the generalizability of these findings is limited due to a restricted number of parents and only a few countries represented in the sample. But the transferability of a qualitative study to another setting is ultimately dependent on the reader (30). We would encourage future research specifically on how interventions could be suited and adapted in order to meet the needs of families with a migration background. This is important since we know that encounters that people with migration background have with healthcare settings (31, 32) could need to be adapted and culturally sensitive in order to meet the needs of the people seeking care. It could also be, that diverse families appreciated the program as the rest of the parents did, since its always the aim of the Swedish healthcare to give equal care to everyone that they meet in all encounters (33).

## Conclusion

The first-time parents involved in this study appreciated the extended home visits that took place when their children were eight months old. Further studies are needed in order to evaluate the effect of these visits on the oral health in the children.

## Data Availability

The raw data supporting the conclusions of this article will be made available by the authors, without undue reservation.
